# Development of a Microheater with a Large Heating Area and Low Thermal Stress in the Heating Area

**DOI:** 10.3390/mi15010130

**Published:** 2024-01-12

**Authors:** Tao Zhang, Zequan Pan, Chunhua Zhang, Liuguang Xiong, Chunmei Yang, Jian Zhang, Mengjiao Shi, Yuhang Wang, Wen Qu

**Affiliations:** 1 College of Electromechanical Engineering, Northeast Forestry University, Harbin 150040, China; zt00663@nefu.edu.cn (T.Z.); m13736023141@163.com (Z.P.); m17763221024@163.com (C.Z.); xiongliuguang328@163.com (L.X.); freezeman007@nefu.edu.cn (J.Z.); 2 Forestry and Woodworking Machinery Engineering Technology Center, Northeast Forestry University, Harbin 150040, China; ycmnefu@126.com; 3 College of Materials Science and Engineering, Northeast Forestry University, Harbin 150040, China; smjsodagreen@163.com

**Keywords:** MEMS, microheater, temperature, large area heating

## Abstract

In this paper, a microheater that can absorb thermal stress and has a large heating area is demonstrated by optimizing the structure and process of the microheater. Four symmetrically distributed elongated support beam structures were machined around the microheater via deep silicon etching. This design efficiently mitigates the deformation of the heated region caused by thermal expansion and enhances the structural stability of the microheater. The updated microheater no longer converts the work area into a thin film; instead, it creates a stable heating platform that can uniformly heat a work area measuring 10 × 10 mm^2^. The microheater is verified to have high temperature uniformity and structural stability in finite element simulation. Finally, thorough investigations of electrical–thermal–structural characterization were conducted. The test findings show that the new microheater can achieve 350 °C with a power consumption of 6 W and a thermal reaction time of 22 s. A scan of its whole plane reveals that the surface of the working area of the new microheater is flat and does not distort in response to variations in temperature, offering good structural stability.

## 1. Introduction

Over the past few decades, dynamic in situ observation techniques have been extensively utilized for the purpose of designing and characterizing materials [1]. In situ observation techniques can provide a more intuitive response to the structure, properties, and reaction principles of materials by providing a continuous process of change under different conditions [2,3]. A number of devices have been developed that can heat samples under vacuum and observe them in real time, such as in situ TEM. Variable-temperature experiments in an in situ TEM allow for the study of the changes in the structure and properties of a material as a function of temperature [4,5,6].

MEMS microheaters offer the benefits of rapid temperature increase and minimal power consumption, making them extensively utilized in in situ observation devices [7,8,9,10]. To enhance the rate at which heat is generated and the upper limit of temperature, it is necessary for these devices to restrict the high-temperature area within a compact volume. Thin-film microhotplates have garnered significant scholarly interest and have undergone rapid development. T. P. Nguyen et al. [11] employed micromachining techniques to create a microheater on a suspended silicon nitride (SiN) film measuring 1 × 1 mm^2^. The microheater exhibited thermal stability up to 500 °C, while mechanical stability of the membrane up to 250 °C was achieved. However, above 250 °C, the film saw a substantial rise in deformation. Woo-Jin Hwang et al. [12] fabricated a novel polycrystalline silicon microheater via power compensation design. The uniform high-temperature area of the microheater was increased by 2.5 times, and the average temperature increased by 40 °C after the power compensation design. Lei Xu et al. [13] successfully created a reliable microheater with minimal power consumption. The power consumption was effectively decreased by employing two elongated beam constructions with a high aspect ratio. The corners of the slender beams were adjusted to minimize heat stress and enhance mechanical robustness.

Although the thin-film architecture has notable benefits, such as decreased power usage and enhanced thermal response, it also has notable drawbacks. The mechanical properties of the thin-film structure are highly unstable and incapable of withstanding heavy samples. During operation, the film experiences internal tensions caused by thermal expansion, which can lead to deformation or even the breaking of the film. In addition, the size of the film is inversely related to its stability, meaning that thin-film microheaters can only offer a limited heating area [14,15,16,17]. Finally, thin-film microheaters are expensive to produce because of the intricate nature of the film manufacturing process. In practical applications, structural stability should be an issue that needs to be focused on. Good mechanical strength can, on the one hand, expand the application range of microheaters, and on the other hand, ensure the stability of the microheater in order to obtain accurate observation data [13]. George Adedokun et al. [18] designed a structurally modified beams suspended membrane microheater with a perforated dielectric layer. This improvement reduces the power consumption of the microheater by approximately 18.6%. Perforated-membrane microheaters have lower thermal stresses in the working area compared to non-perforated membranes. Hotovy, I. et al. [19] fabricated a microheater on a gallium arsenide membrane that was levitated by four cantilever beams oriented diagonally. The mechanical stability of the multilayer membrane structure was tested, and it was found that the center of the microheater deflected more than a few micrometers after heating the microheater plate to a temperature of 350 K. When the microheater was heated to 550 K, the amplitude of the center of the microheater exceeded 30–40 μm. Byeongju Lee et al. [20] prepared an anodized aluminum oxide-based microheater that fabricated a bridge structure with the same thickness as the substrate using the etching mechanism of the anodized aluminum oxide substrate. Because the heating platform uses the entire thickness of the substrate instead of a microscale membrane, the microheater platform has exceptional mechanical and thermal stability.

In this paper, we design a MEMS microheater with a novel support beam structure. Due to the inherent instability of the mechanical structure of thin-film microheaters, we refrain from processing the heating region into a thin film. The new microheater has a larger heating area as well as the ability to carry a larger sample mass. Symmetrically distributed support beams are machined around the working area of the microheater to suspend the working area of the microheater. These support beams are different from the traditional cantilever beam structure, which can better absorb the thermal stresses generated by the heating of the working area. The implementation of this suspended support structure design also diminishes the power consumption of the microheater. In order to enhance temperature consistency, we employ a platinum film with a spiral design that encompasses the entire heating region. Ultimately, we carried out a comprehensive experiment to assess the electrical–thermal performance of the microheater and the structural stability of the microheater while it was in operation.

## 2. Structural Design and Process Flow of Microheater

### 2.1. Structural Design

**Figure 1:**
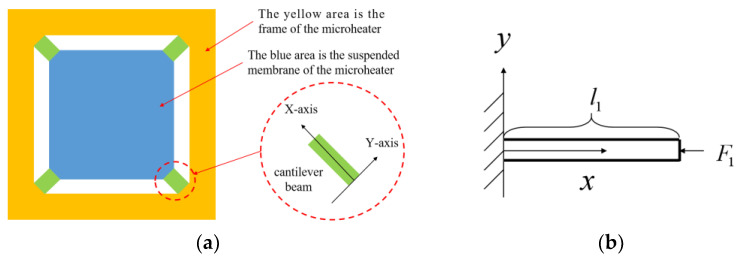
(**a**) Schematic diagram of a miniature heater with conventional cantilever beam construction. (**b**) Schematic diagram of the cantilever beam structure subjected to axial force during the operation of the microheater.

**Figure 2:**
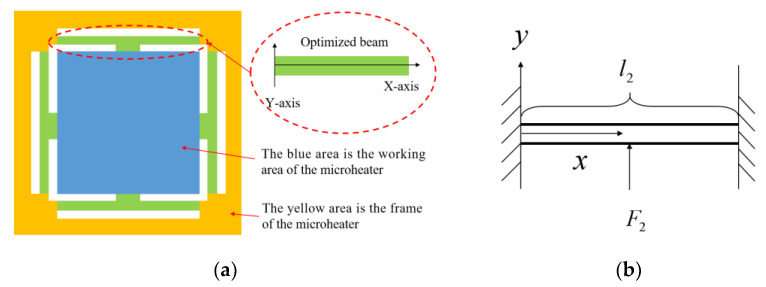
(**a**) Schematic diagram of the microheater with support beam structure designed in this paper. (**b**) Schematic diagram of the force acting on the support beam during the operation of the microheater.

Figure 1a illustrates a typical suspended membrane structure consisting of four diagonally aligned bridge cantilever beams that offer mechanical support for the central suspended membrane. During the operation of the microheater, the central suspended membrane experiences thermal expansion, while the surrounding cantilever beams are subjected to an axial force $F_{1}$ in the direction of the beams, as shown in Figure 1b. In order to ensure the support strength, the length of the thin-film cantilever beam $l_{1}$ is very short (the length is close to the gap between the suspended film and the outer frame, which is usually tens or hundreds of μm); thus, the cantilever beam cannot effectively absorb the expansion caused by the heat of the internal suspended film area. Figure 2a shows the shape of a microheater with support beams after optimization. Instead of a thin-film structure, the new support beams and the central suspended portion retain the silicon substrate, which brings a great enhancement to the mechanical structure stability of the microheater. In contrast to the traditional cantilever beam structure, the novel support beam structure experiences a transverse force $F_{2}$ that is perpendicular to the support beam when the central working zone undergoes thermal expansion, as shown in Figure 2b. Moreover, the length of the support beam structure $l_{2}$ is much larger than $l_{1}$ (the length is close to the size of the working area of the microheater, and the length of the support beam designed in this paper reaches 14 mm) so that the support beam can better absorb the thermal expansion of the working area under the action of the same size of force.

**Figure 3:**
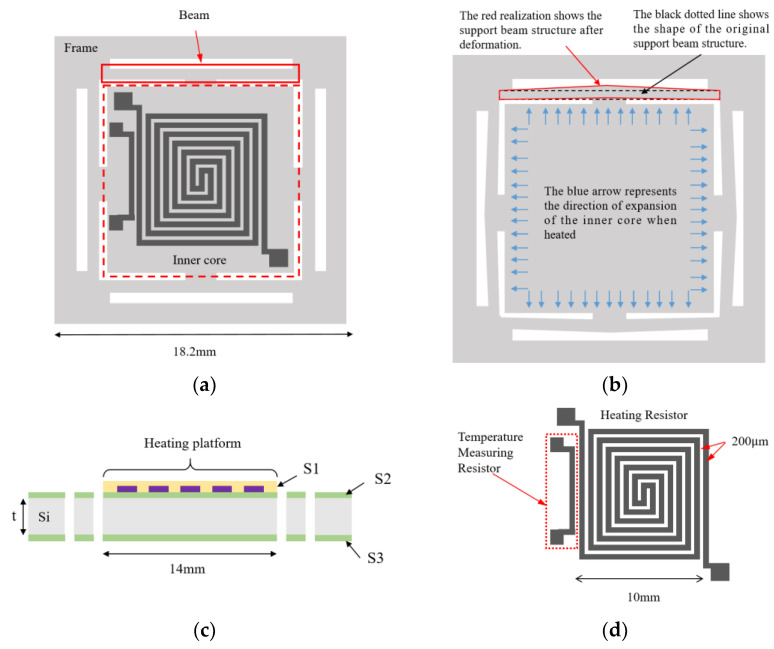
(**a**) Front graphic diagram of microheater. (**b**) Schematic working diagram of support beam. (**c**) Schematic cross-section of a microheater. (**d**) Schematic diagram of heating resistor and temperature measuring resistor.

As shown in Figure 3a, the microheater of the new structure is divided into an inner core and an outer frame by the isolation of the surrounding support beams, with the support beam structure in the red solid box and the inner core region in the red dashed box. When the inner core region expands due to heat, it squeezes the surrounding support beams. These support beams give solid support to the inner core region but do not hinder the expansion of the inner core as in the conventional bridge-type cantilever beam structure. As shown in Figure 3b, the support beams deform when a lateral force is applied perpendicular to them, which greatly reduces thermal stress on the inner core region. The suspended construction has the additional benefit of reducing heat dissipation from the microheater and decreasing power usage.

The heat-generating material for the preparation of microheaters must be characterized by a high melting point, high resistivity, good thermal conductivity, and a small coefficient of thermal expansion [21]. Platinum, tungsten, molybdenum, polycrystalline silicon, and titanium nitride are used as heat-generating materials for microheaters, but each has its own advantages and disadvantages. In recent years, titanium nitride has received particular attention due to its high melting point, good thermal conductivity, mechanical and chemical stability, etc. [22]. Creemer et al. [23] developed a microheater with titanium nitride as a heat-generating material that can operate up to 700 °C but faced the problem of high stress in the titanium nitride film during fabrication. Jithin M.A. et al. [24] investigated the effect of substrate temperature on the properties of titanium nitride thin films and found that the resistivity of titanium nitride thin films decreases with increasing substrate temperature. Platinum is one of the most commonly used heater materials because of its high melting point, good electrical conductivity, stable chemical properties, and excellent mechanical properties [25,26]. Therefore, in this paper, Pt is chosen as the material for heat-generating and temperature-measuring resistors. Figure 3c shows the cross-sectional structure of the microheater. The thermal conductivity of SiO_2_ is relatively poor, so a layer of Si_3_N_4_ with high thermal conductivity is wrapped around the heating resistor on the inner core surface to enhance the temperature uniformity of the inner core. As shown in Figure 3d, in order to further ensure temperature uniformity, the structure of the heating resistor is designed as a double-helix structure. The width of the resistance wire is 200 μm. The temperature measurement resistor is adjacent to the heating resistor and is in the same layer, which serves to measure the surface temperature of the microheater. The main dimensions and materials of the structure are shown in Table 1.

**Figure 4:**
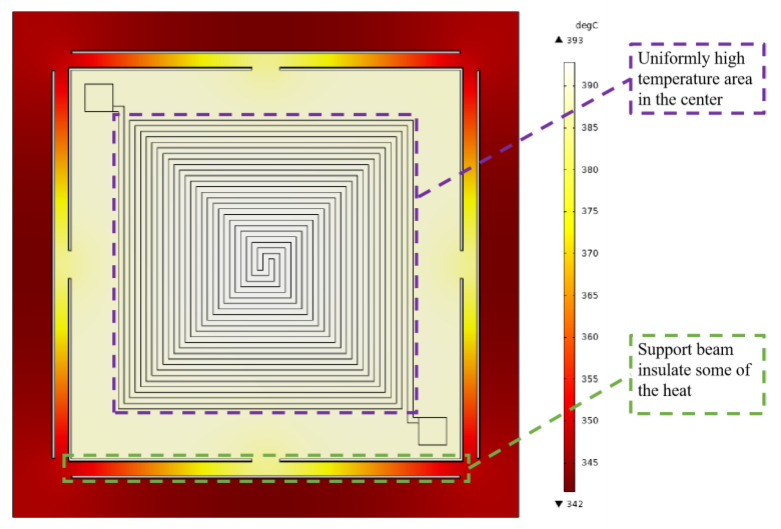
Schematic diagram of temperature simulation results.

**Figure 5:**
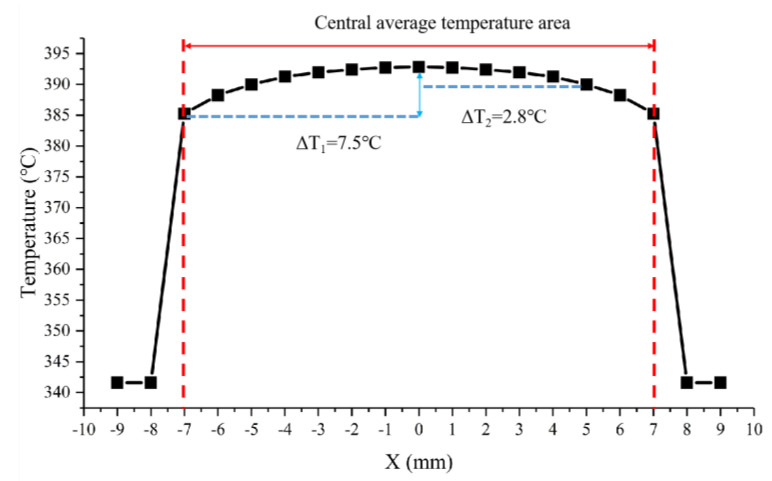
Simulation temperature distribution diagram of microheater along center line.

**Figure 6:**
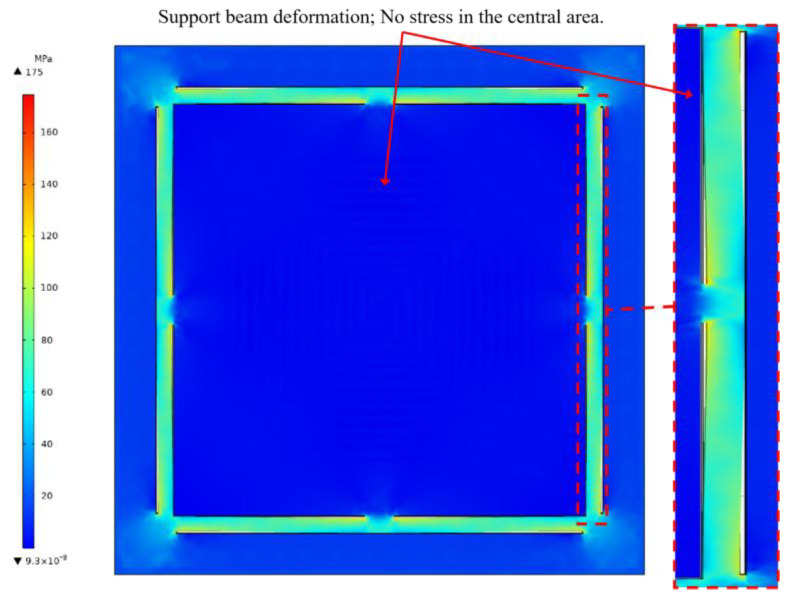
Structural stress simulation diagram of microheater.

We used COMSOL Multiphysics 5.6 simulation software to run coupled electrical, thermal, and structural simulations of microheaters. Specifically, we used the three parts of the software to conduct the simulations, namely, Electric Currents in Layered Shells, Heat Transfer in Solids, and Solid Mechanics. Refer to Table 1 for specific material and size settings for the microheater. To simplify the model, ignore the Pt thin film used only for temperature measurement. First, voltage is set to the heating resistors of the microheater within the Electric Currents in Layered Shells assembly. An operating voltage of 100 V is set at one of the electrodes, and at the other electrode, it is set to ground. Then, the surface heat flux of the microheater is set within the Heat Transfer in Solids assembly with a value of 5 W/(m^2^·K). Finally, within the Solid Mechanics assembly, fixed constraints distributed around the perimeter are added to the microheater. With the above setup, let the COMSOL Multiphysics software solve the model. As shown in Figure 4, the maximum temperature of the center of the microheater at 100 volts can reach 393 °C. From the trend of temperature distribution, it can be seen that the temperature on the support beam shows a significant downward trend, so the support beam plays the role of thermal insulation and reduces the loss of heat. Taking the center of the microheater as the origin and the centerline along the horizontal direction as the *x*-axis, the temperature distribution of the microheater along the centerline is shown in Figure 5. As can be seen from the blue comparison line in the figure, the microheater working area has good temperature uniformity, with a maximum temperature difference of 7.5 °C in the range of 14 × 14 mm^2^ at the center and a maximum temperature difference of 2.8 °C in the range of 10 × 10 mm^2^. The results of the microheater stress simulation are shown in Figure 6. From the enlarged view of the details circled by the red dashed box, it can be seen that the deformation of the surrounding support beams absorbs the thermal stresses generated by the heating of the working area and ensures the stability of the working area.

### 2.2. Process Flow

**Figure 7:**
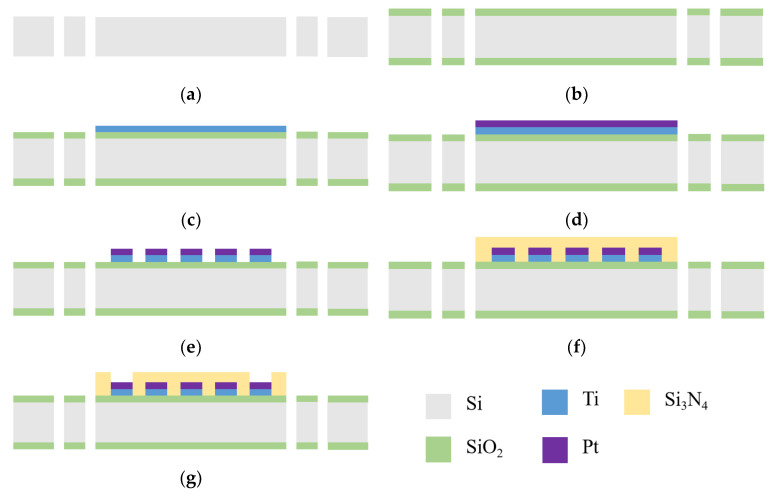
The production process of microheaters: (**a**) deep silicon etching processing support beam; (**b**) deposited SiO_2_; (**c**) magnetron-sputtered Ti thin films; (**d**) magnetron-sputtered Pt thin films; (**e**) patterned Pt/Ti; (**f**) deposited Si_3_N_4_; (**g**) open a window at the wire bonding point.

The overall processing flow of the microheater is shown in Figure 7. First, deep silicon etching was used to machine through holes on the surface of a 500 μm thick double-thrown silicon wafer to form support beams. Then, a SiO_2_ layer with a thickness of 500 nm was deposited on both sides of the wafer using thermal oxidation. Because Pt and SiO_2_ do not stick together very well, 50 nm Ti was sputtered on as an adhesion layer via reactive magnetron sputtering. This was followed by 200 nm thick Pt being sputtered on. Ion etching was then used to make the temperature measurement resistor and spiral resistor wire into the shape that was wanted. Then, a 300 nm thick layer of Si_3_N_4_ was deposited on the surface of the inner core area using PECVD. The Si_3_N_4_ layer is 100 nm thicker than the Pt layer underneath to ensure complete insulation and to protect the resistor wires from environmental influences. The excess silicon nitride was then etched away in the pad area using the RIE technique, exposing the underlying Pt to facilitate lead bonding. Finally, individual microheaters were cut from the wafer.

**Figure 8:**
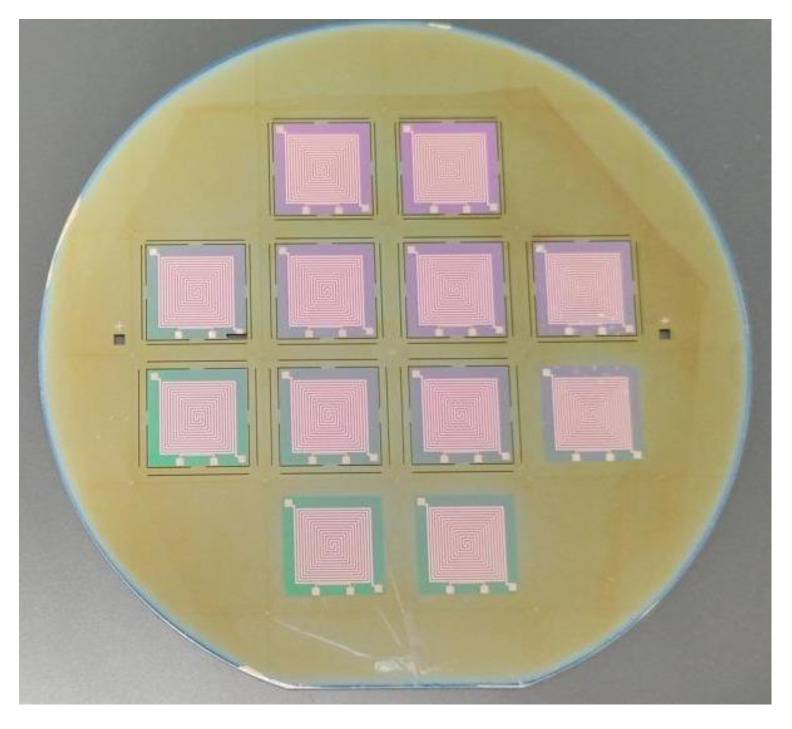
Microheaters obtained by the process.

**Figure 9:**
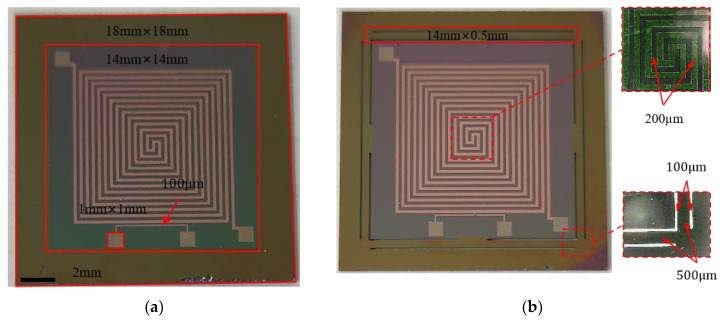
Microheater image after slicing: (**a**) Structure 1—unoptimized microheater; (**b**) Structure 2—microheater with supporting beams.

According to the aforementioned process, we successfully produced 12 microheaters on a 4-inch wafer, each with a substantial heating surface area, as shown in Figure 8. From the illustration, it is evident that all the remaining microheaters are in pristine condition, except for one microheater inner core that has sustained damage to one of its corners during long-distance transportation. We performed a basic power-on test on all the processed microheaters, and the outcomes indicated that all the microheaters functioned correctly. To make the experimental results comparable, an unoptimized planar microheater (Structure 1) and an optimized microheater with a support beam structure (Structure 2) were fabricated using the same process. The optical images of the machined individual microheaters are shown in Figure 9, we have circled some of the important structures in the microheater with red wireframes and labeled the dimensions next to them, while the enlarged view is circled with a red dashed wireframe.

## 3. Electrical–Thermal–Mechanical Structural Characteristics of Microheater

In this chapter, we performed a complete electrical–thermal–mechanical structural characterization of the microheater. First, we tested the electrical–thermal performance of the microheater. The power consumption temperature profile, the temperature resistance coefficient of resistance of the measured temperature, and the rate of warming and cooling were determined by heating the microheater. The maximum temperature that the microheater can reach was tested. Finally, the deformation that occurs in the working area of the heated microheater was investigated with respect to the stability of the mechanical structure.

### 3.1. Electrical–Thermal Characteristics

**Figure 10:**
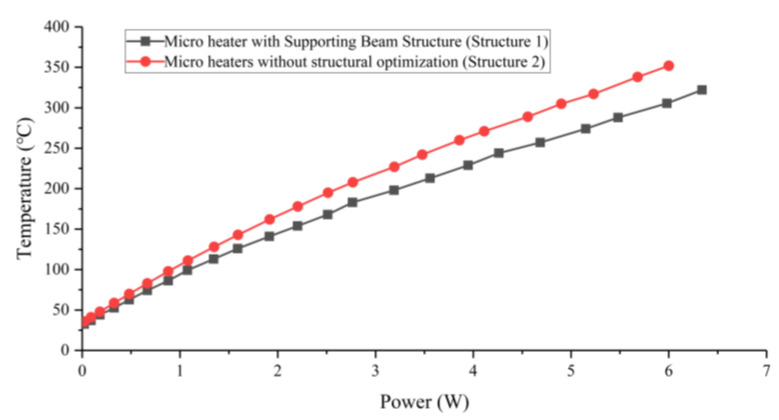
Microheater power–temperature curve.

We analyzed the electrical–thermal performance of the microheater. The experimental setup has four components: a microheater, a programmable DC power supply, an infrared camera (Testo 890), and a workstation for control and documentation. The wire-bonding process is used to bond the leads on the heating resistor and temperature measuring resistor electrodes for subsequent wire connections. The measured temperature and power relationship, applying a DC voltage to the microheater heating resistor through a DC power supply, are shown in Figure 10. Based on the graph, it is evident that Structure 2 demonstrates significantly superior power consumption characteristics. When the input voltage is 100 V, Structure 2 reaches 350 °C with 6 W of heating power. In contrast, the power consumption of Structure 1 with the same access to 100 V reaches 6.3 W, while the temperature only reaches 322 °C. Thus, the structural design of the support beam designed in this paper can provide good thermal insulation and reduce the power consumption of the microheater. It should be noted that the processed platinum film is not absolutely uniform, and the measurements were not performed under vacuum conditions with uncontrolled convective heat dissipation between the microheater and the environment. As a result, there are some deviations between the experimentally measured temperatures and the simulation results, but in general, they are within acceptable limits.

**Figure 11:**
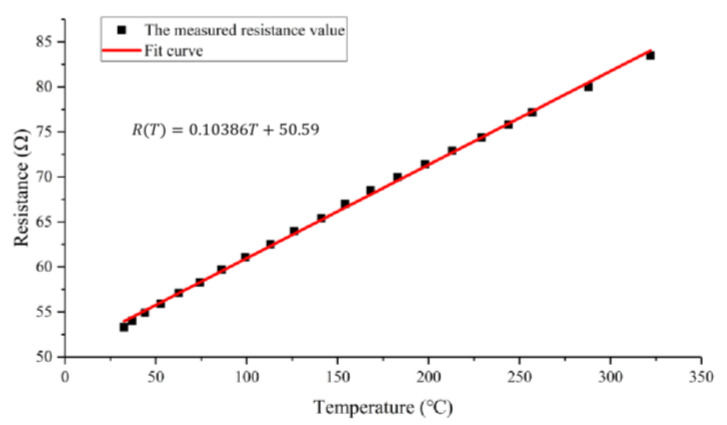
Temperature–resistance curve of microheater measuring resistance.

The principle of metal thin film temperature measurement is that when the temperature rises, its resistance also rises, and Pt has a good linearity of temperature resistance coefficient. Therefore, it is possible to respond to changes in temperature based on changes in resistance [27,28]. We used an almost linear fit to the measured temperature and the temperature resistance value to find the temperature coefficient of resistance (TCR). Figure 11 shows the relationship between resistance and temperature, which shows that the temperature–resistance curve is highly linear. TCR can be calculated using the following equation:

(1) $$\alpha =\left(R_{T}-R_{0}\right)/[R_{0}\left(T-T_{0}\right)]$$

where α represents the TCR value, and R_0_ represents the resistance at the initial temperature T_0_. In this paper, the TCR value of the temperature measurement resistance of the microheater is calculated to be 0.00205 K^−1^.

**Figure 12:**
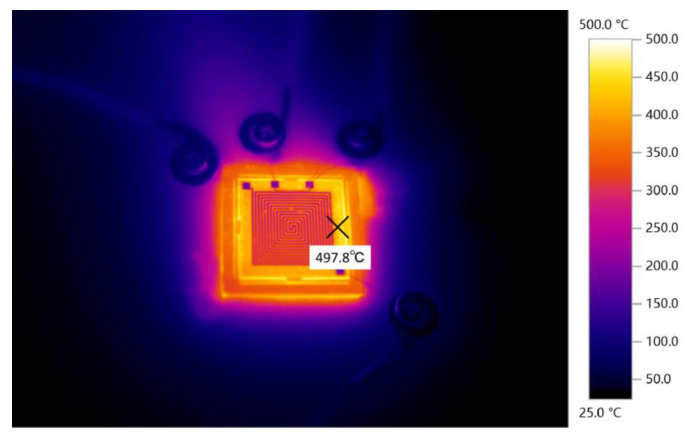
Infrared imaging of a microheater.

The main heating range of our microheater is from room temperature to 350 °C, but some special organic materials have a melting point of about 500 °C. Therefore, we subjected the microheater to further high-temperature testing. The voltage was brought to 155 V by controlling the DC power supply, at which time the power consumption of the microheater came to 9.1 W, while the temperature of the microheater increased to 497.8 °C. Figure 12 shows a thermal image of the microheater at this temperature. The microheater was tested at that temperature without any warping or cracking of the surface.

**Figure 13:**
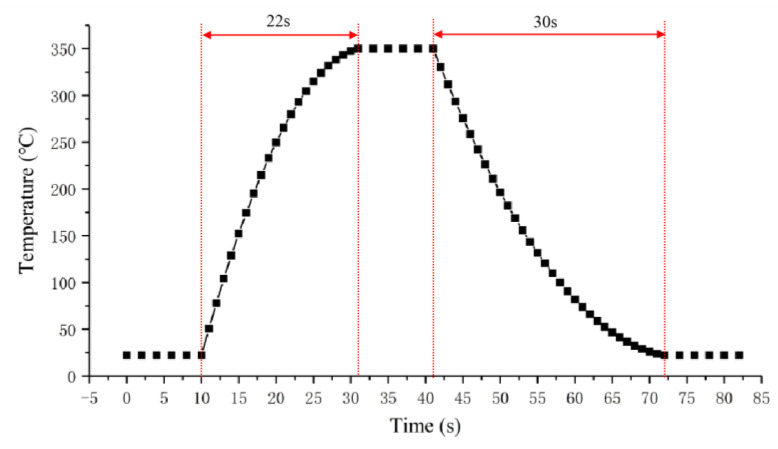
Heating and cooling curves of microheaters.

The rate of warming of Structure 2 was tested at a safe temperature of 350 °C. The current temperature of the microheater is detected by the previously calibrated temperature measuring resistor. Similarly, the cooling rate of the microheater was tested after turning off the power. The heating and cooling curves of the microheater are shown in Figure 13. The test results show that the microheater heats up at an average rate of 14.9 °Cs^−1^ and cools down at an average rate of 10.3 °Cs^−1^.

### 3.2. Electrical–Thermal Characteristics

**Figure 14:**
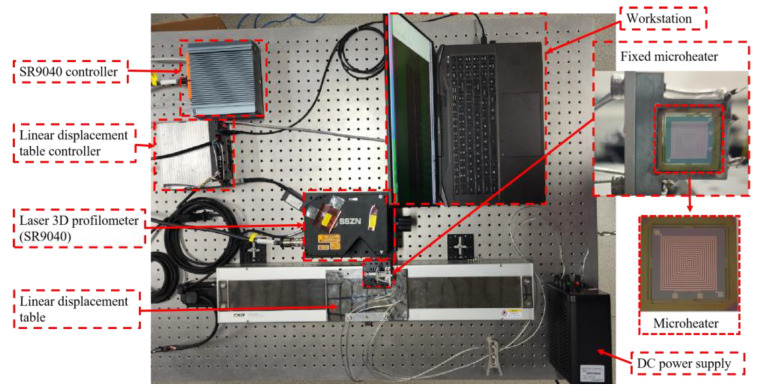
Images of experimental instruments used for 3D contour scanning.

**Figure 15:**
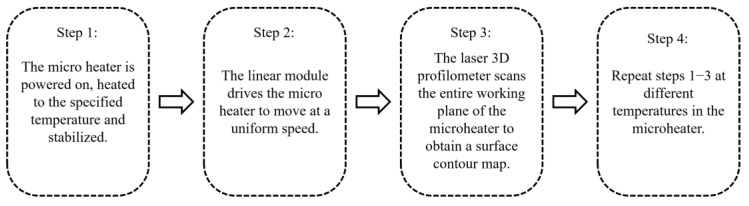
Experimental flowchart.

To confirm the enhanced structural stability of the microheater with the support beam structure proposed in this study during operation, we conducted measurements of the deformation of Structure 1 and Structure 2 under heating conditions, and the experimental setup is shown in Figure 14. The whole experimental system consists of five parts: a fixed MEMS microheater, a laser 3D profilometer, a fully enclosed type-slide module driven by a motor, a DC power supply, and a workstation for control and recording. Figure 15 shows the whole experimental flow. First, the microheater fixed on the linear module is heated by the DC power supply, and the temperature of the microheater is measured by the temperature measuring resistor. After the temperature is stabilized, the linear module drives the fixed microheater to do horizontal displacement so that the entire plane of the microheater passes through the beam of the laser 3D profiler. The laser 3D profiler scans the working area of the heater, and the surface profile of the heater is obtained by processing the measured point cloud data. The experimental procedure was repeated at different temperatures to obtain the deformation profiles and data of the microheater at different temperatures. In order to ensure the stability of the test process, the entire test system is placed on a pneumatic platform.

**Figure 16:**
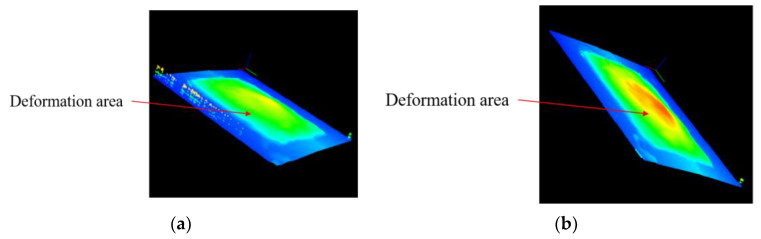
(**a**) 3D contour map of Structure 1 at 100 °C; (**b**) 3D contour map of Structure 1 at 350 °C.

**Figure 17:**
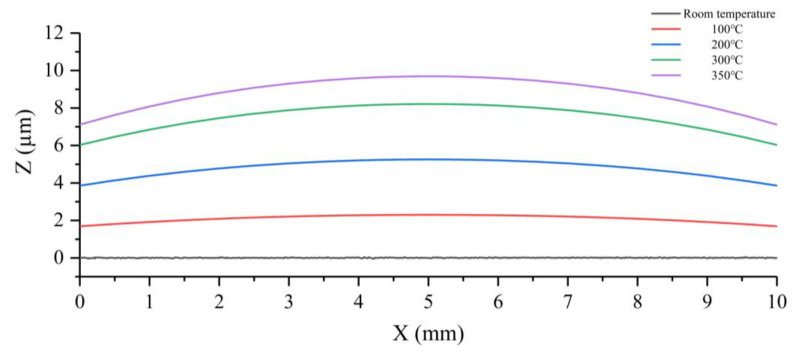
Two dimensional cross-sectional profile curves of Structure 1 in the working area at different temperatures.

Figure 16a shows a 3D contour map of Structure 1 heated to 100 °C, and Figure 16b shows a 3D contour map of Structure 1 heated to 350 °C. The blue to red color in the figure indicates that the deformation increases sequentially. It can be seen from the figure that the center region of Structure 1 undergoes a raised deformation during the heating process, where the largest deformation is located in the center of the microheater. After several measurements, the two-dimensional cross-section profile curves of the center working region of Structure 1 at different temperatures are shown in Figure 17. It can be seen that the higher the temperature, the larger the bulge at the center of the microheater. The microheater center deformation reaches a maximum of 9 μm at a temperature of 350 °C.

**Figure 18:**
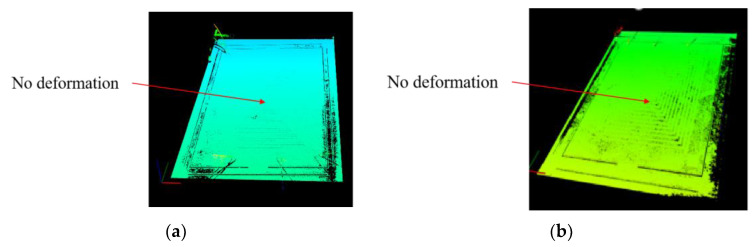
(**a**) 3D profile of Structure 2 at 100 °C; (**b**) 3D profile of Structure 2 at 350 °C.

**Figure 19:**
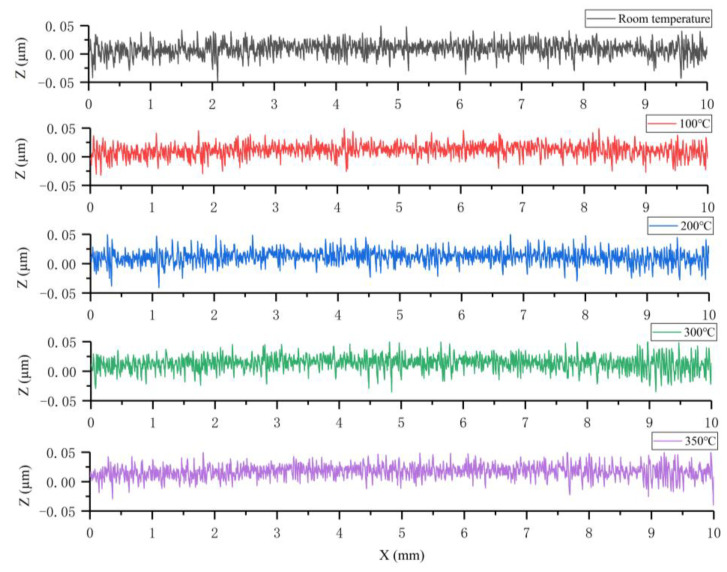
Two dimensional cross-sectional profile curves of Structure 2 in the working area at different temperatures.

Similarly, Structure 2 was heated and scanned, and the resulting 3D contour map is shown in Figure 18. It can be seen that the center working area of Structure 2 still maintains an intact surface flatness after warming up, as the colors in the 3D contour map are essentially the same. The two-dimensional cross-section profile curves of the working region of Structure 2 at different temperatures are shown in Figure 19, from which it can be seen that the cross-section of Structure 2 still has good linearity at high temperatures without large deformation. This indicates that the support beam structure designed in this paper can well absorb the expansion of the center working area due to heat, which enhances the mechanical–structural stability of the microheater.

## 4. Conclusions

This paper investigates the design and fabrication of a MEMS microheater with a support beam structure. The microheater is characterized by a large heating area and low thermal stress in the heating region. We used COMSOL to simulate the microheater’s temperature and stress distribution after it was turned on. The simulation results show that the microheater has a highly uniform surface temperature and that the support beam does a good job of absorbing thermal stress in the heating area.

A microheater with a heating area of 10 × 10 mm^2^ was obtained by using bulk micromachining technology and surface micromachining technology. The machining process and fabrication procedure of the MEMS microheater are introduced, and a microheater with a heating area of 10 × 10 mm^2^ is obtained. We tested the electrical and thermal characteristics of the microheater in detail. The experimental results show that the microheater designed in this paper has a very good heating efficiency, requiring only 6 W of heating power to warm up the heating area to 350 °C, and the heating area has good temperature uniformity. The average heating rate of the microheater is 14.9 °C, and the average cooling rate is 10.3 °C. Furthermore, the capacity of the support beam structure to mitigate thermal stresses in the operational region of the microheater was confirmed. A laser 3D profilometer was employed to scan the operational region of the microheater. The experimental findings demonstrate that the microheater’s working area, when equipped with the support beam structure, consistently maintains a flat configuration without any deformation throughout varying temperatures. The support beam construction proposed in this research can significantly enhance the structural stability of the microheater.

## Figures and Tables

**Table 1:** Materials and structural dimensions of microheaters.

	Material	Thickness (nm)
Membrane	Si_3_N_4_ (layer S1)	S1 = 300
	SiO_2_ (layer S2)	S2 = 500
	SiO_2_ (layer S3)	S3 = 500
Heating resistor	Ti + Pt	250
Temperature measuring resistor	Ti + Pt	250
Chip	Si	t = 5 × 10^5^

## Data Availability

Data are contained within the article.
